# Awake Craniotomy for a Frontal Astrocytoma: A Case Report

**DOI:** 10.7759/cureus.59667

**Published:** 2024-05-05

**Authors:** Vladislav Velchev, Stefan Burev, Dilyan Ferdinandov, Deyan Popov, Petra Vasileva, Stela Petrova, Petar-Preslav Petrov, Remzi R Hyusein, Plamen Penchev

**Affiliations:** 1 Faculty of Medicine, Medical University of Plovdiv, Plovdiv, BGR; 2 Department of Neurological Surgery, University Hospital "St. Ivan Rilski", Sofia, BGR; 3 Department of General and Clinical Pathology, University Multi-profile Hospital for Active Treatment and Emergency Medicine (UMHATEM) - Pirogov, Sofia, BGR; 4 Department of Anatomy, Histology, and Embryology, Medical University of Plovdiv, Plovdiv, BGR; 5 Faculty of Medicine, Medical University of Sofia, Sofia, BGR

**Keywords:** astrocytoma, low-grade gliomas, awake craniotomy, neuronavigation guidance, frontal tumour, case report

## Abstract

Awake craniotomy is a surgical procedure that has been gaining significance over the past decades. Neuronavigation is an intraoperative technology that locates tumors and monitors the brain cortex during awake craniotomy. The presence of cerebral low-grade gliomas in the frontal lobe creates a risk of affecting vital centers of the brain cortex during surgery. We present a clinical case of a 42-year-old male patient who entered the neurosurgery clinic with a clinical manifestation of headache for two months. MRI showed evidence of the recurrence of a left frontal glioma. Differential diagnoses of frontal gliomas include metastases, abscesses, and cysts. The pathophysiologic background of the disease is the mutation of neuroglial cells, which leads to an abnormal and uncontrollable proliferation. Under sleep-awake anesthesia, operative treatment was performed through left frontal awake craniotomy under neuronavigation. As a result, a total excision was achieved. Motor functions of the right limbs and speech have been preserved. The patient was mobilized on the day after the intervention. Surgery-related complications were not observed. The patient had relief from the symptoms and was discharged on the fifth day. Awake craniotomy combined with neuronavigation was the most efficient and the least harmful method for the excision of the tumor. For low-grade gliomas localized in the frontal area of the encephalon, awake craniotomy is the only secure option for surgery.

## Introduction

Awake craniotomy is an innovative method broadly used in contemporary neurosurgical operations due to its efficacy for complicated cases affecting the cerebrum. It is performed under tightly controlled anesthesia switching stages of full consciousness and anesthetic episodes. This manipulation is critically implemented especially for patients with lesions located within or near functionally vital motor, cognitive, or cortical regions, such as those in the frontal lobe [1].

The standard procedure for awake craniotomy is performed while the patient is conscious, with electrical stimulation brain mapping or neuronavigation. The patient is initially put under sleep-awake anesthesia. Incisions of the skull and the meninges are made, which create access to the brain structures. Local anesthesia at the scalp is added and the patient is brought back to consciousness [2]. Direct electrical stimulation is used to identify and map the functional regions around the zone of the incision. After the vital cortical parts have been identified, the tumor is removed [3].

The precision of locating the tumor and the affected cerebral regions, as well as the position of the surgical instruments, is accomplished through live neuronavigation [4]. Frameless stereotactic neuronavigation, an intraoperative technology that locates tumors and monitors the brain cortex during awake craniotomy, is currently a predominant tool in planning a surgical approach for resection [5].

Gliomas are a broad group of central nervous system tumors originating from the glial cells. They have been histologically classified according to the 2021 World Health Organization Classification of Tumors of the Central Nervous System as astrocytomas, oligodendrogliomas, and glioblastomas [6]. Such formations could develop in all lobes of the brain; however, 40% of cerebral low-grade gliomas appear in the frontal lobe. The presence of essential centers in the frontal lobe such as Broca’s and motor function areas creates a hazard during craniotomy and tumor extirpation. Therefore, awake craniotomy and neuronavigation are methods widely used for frontal lobe surgeries [7].

The aim of the report is to present a clinical case of frontal astrocytoma surgically extirpated through awake craniotomy using brain mapping and to evaluate the application of the method for gliomas occurring near essential centers of the cortex.

## Case presentation

We present a clinical case of a 42-year-old male patient who entered the neurosurgery clinic with a clinical manifestation of headaches for two months. Medical history included a previous tumor formation in the left frontal region with histological evidence for a second-grade astrocytoma that occurred two years ago. The patient has been treated with chemotherapy and was regularly examined with MRI.

**Figure 1:**
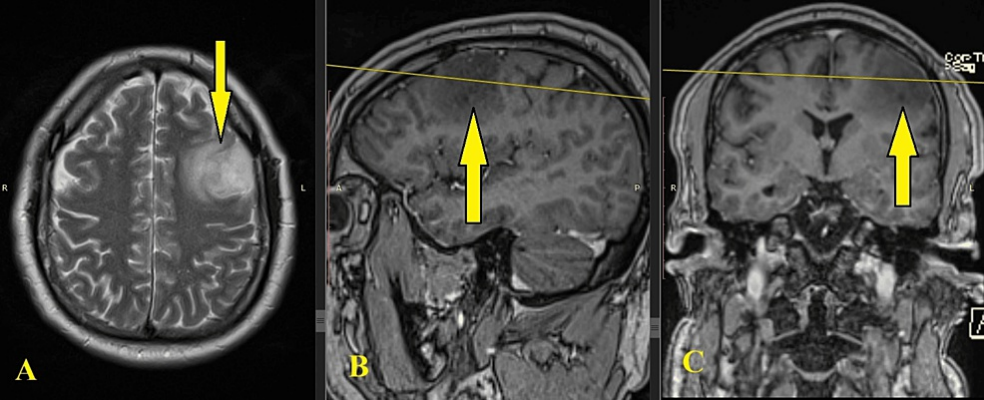
Preoperative MRI of left frontal glioma. (A) Axial plane; (B) sagittal plane; (C) coronal plane. Notice the tumor formation in the left frontal region of the cerebrum.

On the last follow-up, an MRI showed evidence of the recurrence of the left frontal glioma (Figure 1). After consultation with a neurosurgeon, the patient entered the clinic for surgical treatment.

**Figure 2:**
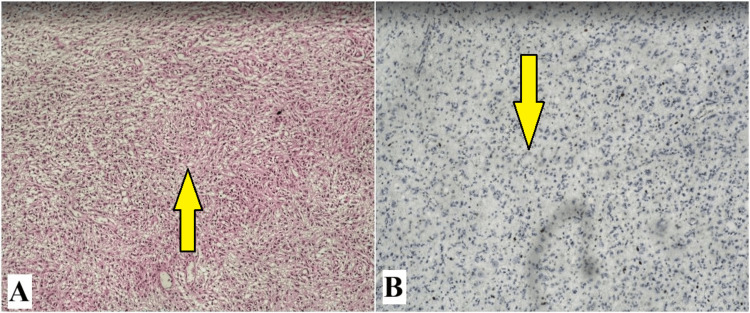
Cerebral cortex in places with infiltration from astroglial tumor with diffuse growth and well-defined neuropil. Minimal cellular and nuclear atypism. Poorly expressed vascular proliferation. Absence of necrosis and hemorrhage. The mitotic index (Ki 67) is less than 4%. (A) Cerebral cortex; (B) white matter.

The histological results discovered an astroglial tumor with diffuse growth and well-defined neuropil characterized by minimal cellular and nuclear atypia, poorly expressed vascular proliferation, and absence of necrosis and hemorrhage. An immunohistochemical test with a Ki 67 marker was performed, which showed less than 4% mitotic activity (Figure 2).

**Figure 3:**
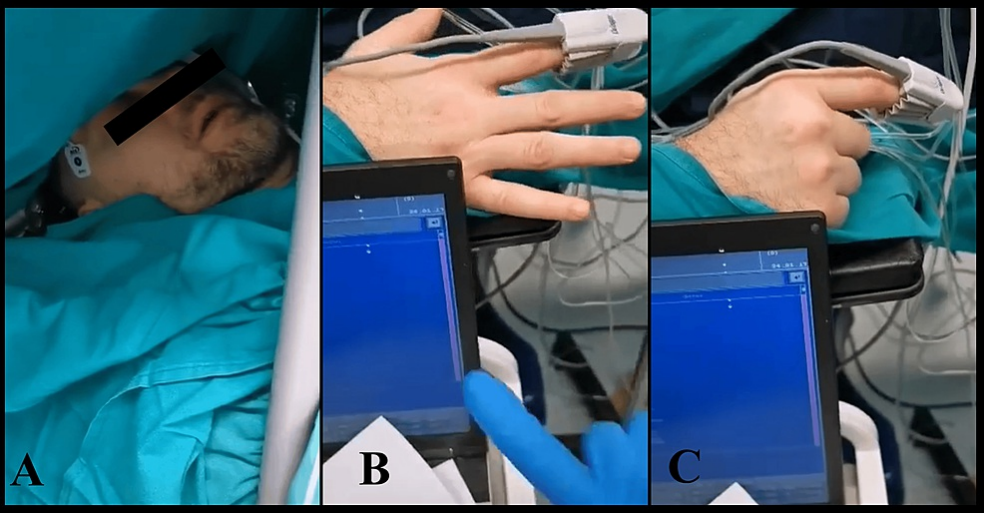
Intraoperative photography. (A) Awake craniotomy with sleep-awake anesthesia. (B & C) Evaluation of the fine motor skills of the right upper limb of the patient.

**Figure 4:**
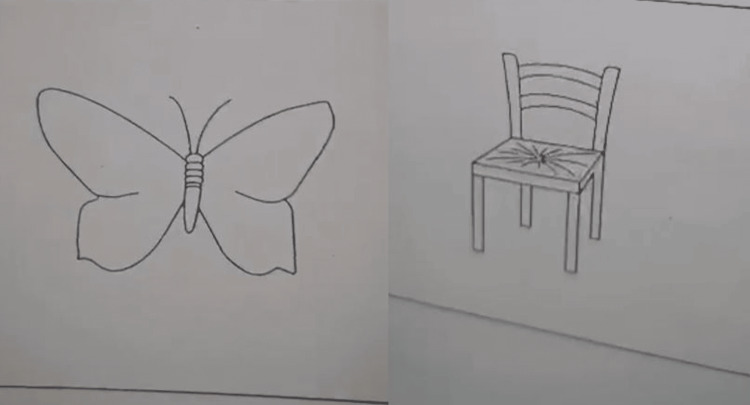
Materials (pictures) used for evaluating the cognitive skills of the patient during awake craniotomy of the left frontal region.

Operative treatment was performed through awake craniotomy. The patient was positioned on the operating table and the operational field was prepared. Access to the cerebrum was provided through left frontal craniotomy under sleep-awake anesthesia. Then, the patient was awakened and an extirpation of the tumor mass was done under precise neuronavigation control. The fine motor skills of the right upper limb have been monitored (Figure 3). The patients’ cognitive skills have been evaluated by picture identification and associations (Figure 4).

**Figure 5:**
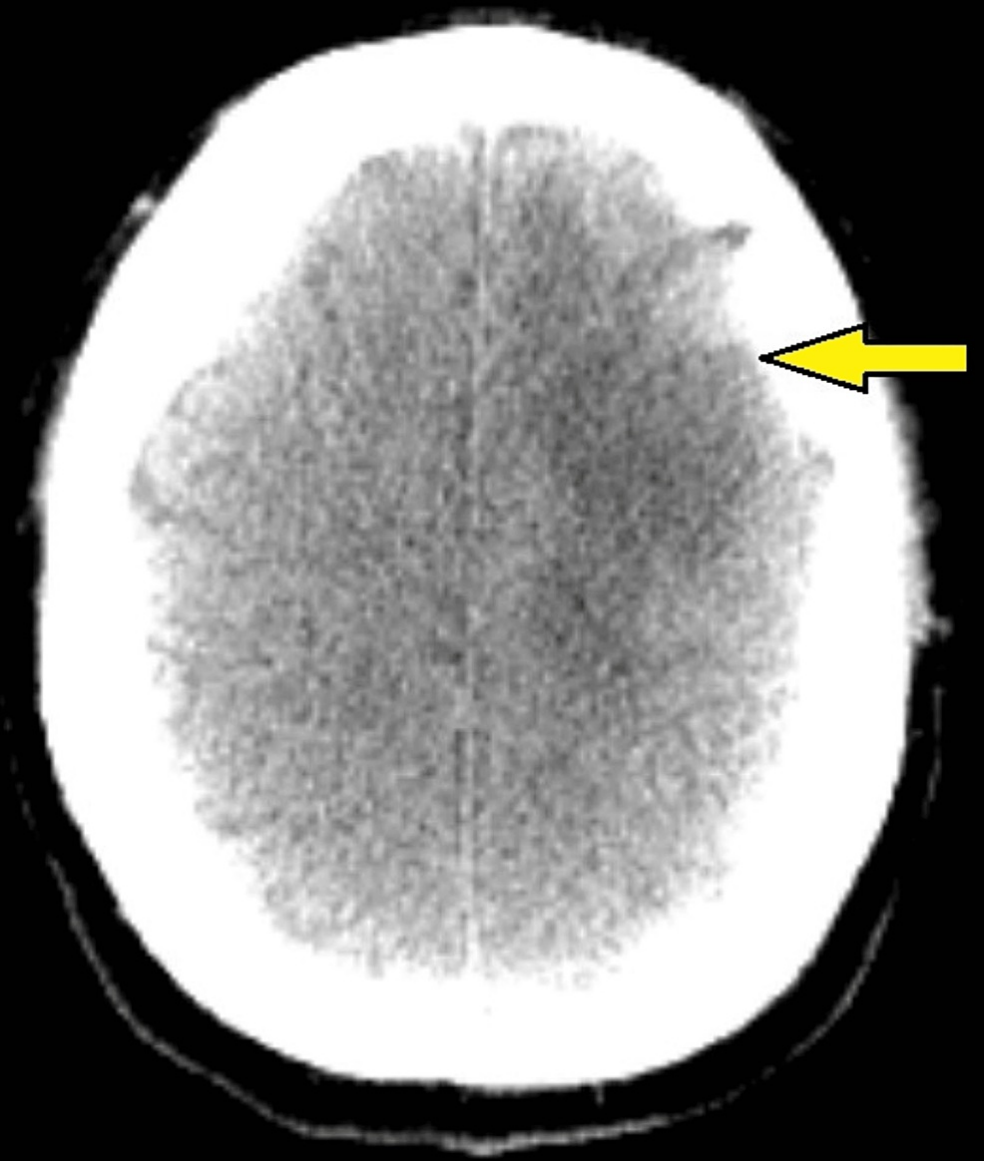
Postoperative CT after surgical exertion of a left frontal glioma. Axial plane. Notice the exerted part in the left frontal region of the cerebrum.

As a result, a total extirpation of the tumor mass was achieved (Figure 5). Motor functions of the right limbs and speech have been preserved. The patient was mobilized on the day after the intervention. Surgery-related complications were not observed. The patient had relief from the symptoms and was discharged on the 5th day.

## Discussion

Astrocytic gliomas are the most frequent intracranial tumors [8]. The pathophysiologic background is the mutation of neuroglial cells, which leads to abnormal and uncontrollable proliferation. The cancerous cells prefer abnormal energy generation through aerobic glycolysis and exhibit a natural resilience to apoptosis. This process is commonly accompanied by rapid angiogenesis. The fast enlargement of the formation could lead to intracranial hemorrhage in some cases [9]. Histological examination of the patient’s biopsy discovered an infiltration from the astroglial tumor with diffuse growth and well-defined neuropil of the cerebral cortex and white matter. Minimal cellular and nuclear atypism has been found. Vascularization was poorly expressed, and hemorrhages were absent. The mitotic index (performed with Ki 67) was less than 4%. This description matches the pathophysiological description of low-grade astrocytomas. Differential diagnoses of gliomas include metastases, abscesses, and cysts [7].

As the frontal lobe is established late in cranial development, it is the core of advanced cognitive functions such as language, emotions, memory, and decision-making [10]. Frontal tumors could create impairments in cognitive functions. Surgical interventions in such vulnerable regions in communication with Broca’s and motor function areas could be exceptionally threatening as the functionality of the brain could be damaged. Bakheit et al. have documented a rare disorder, anarchic hand syndrome, which has been induced by the invasive exertion of a frontal glioma [11].

To prevent complications, methods broadly used to manage frontal lobe gliomas are awake craniotomy and brain mapping through neuronavigation. The precise cooperation between the neurosurgeon and the anesthesiologists is essential [12]. The predominant approach for sedation during awake craniotomy is the systemic administration of anesthetic drugs in strictly controlled amounts [13]. Our patient’s treatment followed these established principles.

This method has been exceptionally successful for numerous patients with tumors near eloquent cerebral regions. Mackel et al. have effectively extirpated a cancerous mass of an awake patient who was kept conscious and performed music to assess the cognitive functions during the operative process [14]. Alternatively, Brosnan et al. performed an asleep-awake-asleep craniotomy on a non-verbal patient by the use of neuronavigation and 5-aminolevulinic acid fluorescence, and postoperative examination proved an improvement in speech and cognition [15]. Bolzani et al. concluded that although the maintenance of analgesia and hemodynamic stability is a challenge with an awake patient, the target-controlled infusion of anesthetic drugs can provide the needed level of consciousness, which is essential for the satisfactory outcome of the procedure [16].

Based on the unappealable evidence for the success of awake craniotomy and neuronavigation, the patient’s treatment was performed in the asleep-awake-asleep principle. Utmost extirpation has been achieved and meticulous hemostasis was accomplished. Motor functions of the right limbs and speech have been completely preserved. Neuronavigation provided a deeper scope into the functional areas of the cerebrum. As no damage has been determined, the method was assessed as the most efficient way of managing the frontal astrocytoma. However, regular follow-up examinations are compulsory to prevent major recurrence and deterioration of the patient’s condition [17].

## Conclusions

The majority of cerebral low-grade gliomas appear in the frontal lobe, which creates а risk of affecting eloquent regions of the brain, such as Broca’s and motor function areas. Awake craniotomy supported by advanced methods of neuronavigation is the most effective and the least harmful option for patients with low-grade gliomas localized in the frontal area. The precise cooperation between the neurosurgeon and the anesthesiologists is of great importance for the success of the procedure. Follow-up examinations preventing the recurrence of the glioma are compulsory.
